# Acupotomy for calcaneodynia: A systematic review protocol

**DOI:** 10.1097/MD.0000000000010143

**Published:** 2018-04-06

**Authors:** Yifeng Shen, Qiaoyin Zhou, Zuyun Qiu, Yan Jia, Shiliang Li

**Affiliations:** Department of Acupuncture-Moxibustion, China-Iapan Friendship Hospital, Beijing, China.

**Keywords:** acupotomy, calcaneodynia, protocol, systematic review

## Abstract

**Background:**

Calcaneodynia customarily induces inferior heel pain, specialists believe that the pain is principally caused by acute or chronic injury due to the plantar fascia from accumulative overload pressure. Acupotomy has been widely used to treat calcaneodynia. But its efficiency has not been scientifically and methodically evaluated. The aim of this study is to evaluate the efficacy and safety of the acupotomy treatment in patients with calcaneodynia.

**Methods:**

Relevant randomized controlled trials in 6 databases (PubMed, Embase, and Cochrane Library, Chinese literature databases, Chinese Biomedical Literature Database [CBM], China National Knowledge Infrastructure [CNKI], and Wanfang Database). The randomized controlled trials (RCTs) of the acupotomy for calcaneodynia patients will be searched in the databases from inception to December 2017 by 2 researchers. Visual analog scale (VAS) will be assessed as the primary outcomes. Roles and Maudsley Score (RM) will be assessed as the secondary outcome. The RevMan V.5.3 will be used for meta-analysis. Continuous outcomes will be presented as the mean difference or standard mean difference, while dichotomous data will be expressed as relative risk.

**Results:**

This study will provide a high-quality synthesis of VAS and RM to assess the effectiveness and safety of acupotomy for calcaneodynia patients.

**Conclusion:**

This systematic review will provide evidence to judge whether acupotomy is an effective intervention for patients with Calcaneodynia.

## Abstract 2

Supplemental Digital Content is available in the text

## 1 Introduction

Calcaneodynia or heel pain, a common clinical complaint, is mainly caused by bony or soft-tissue disorders.^[1,2]^ These disorders are classified according to their anatomical origin and predominant location of heel pain, including plantar fascial lesions, heels spurs, heel plantar fat pad abnormalities, tendinous lesions, Haglund disease, and bursal lesions. Specialists believe that the pain is principally caused by acute or chronic injury due to the plantar fascia from accumulative overload pressure.^[3,4]^ Most of the patients cannot stand or walk for a long time, the pain may be more severe during the first minutes of rest after a walk.^[5,6]^ Calcaneodynia occurs in up to 10% of the population, the condition affects active and sedentary adults of all ages.^[7]^

Most interventions used to manage calcaneodynia have not been adequately studied; however, shoe inserts, stretching exercises, steroid injection, and custom-made night splints may be beneficial.^[8–12]^ Extracorporeal shock wave therapy may be effective in treating patients with chronic heel pain but is ineffective in others.^[13]^ Limited evidence^[14]^ suggests that casting or surgery may be beneficial when conservative measures fail.

Acupotomy is a miniature surgery instrument, consists of handle, needle body and blade.^[15]^ It could cut and detach the abnormal, cicatricial, and contractured tissues with microtrauma.^[16]^ Acupotomy has been widely used clinically by doctors of traditional Chinese Medicine, orthopedics and pain department to treat calcaneodynia in China with satisfied efficacy.^[17–21]^ Some trials^[22,23]^ reported the acupotomy could effectively alleviate pain symptoms, change the thickness of the plantar fascia, attenuate the plantar stress, and benefit for recovery of the normal force structure of patients’ feet.

This study adopts the method of evidence-based medicine to analyze and evaluate clinical RCTs in patients with calcaneodynia, in order to provide evidence for further enhancing the clinical curative effect on patients with calcaneodynia. The study will assess the effectiveness and safety of the acupotomy treatment in calcaneodynia patients.

## 2 Methods

### 2.1 Inclusion criteria for study selection

#### 2.1.1 Types of studies

All the RCTs of acupotomy for the management of calcaneodynia patients will be included without publication status restriction or writing language.

#### 2.1.2 Types of patients

Studies involving patients of any age with calcaneodynia will be included without limitations related to gender, race, and education status. Fracture and dislocation, muscle injury, osteomyelitis, bone tuberculosis, bone tumors, and other causes of calcaneodynia will be expelled.

#### 2.1.3 Types of interventions

##### 2.1.3.1 Experimental interventions

The treatment group will be treated with acupotomy (there is no limit on the needle materials, treatment methods, and course of treatment).

##### 2.1.3.2 Control interventions

Because there is no false acupotomy reported in the literature and acupotomy commonly used in the acupuncture-moxibustion department. The control group will adopt the internationally recognized therapy such as block therapy or no treatment, acupuncture will also be included. Acupotomy with another active therapy versus the same therapy alone will also be investigated. Studies comparing 2 different types of acupotomy or surgical procedures will be expelled.

#### 2.1.4 Types of outcome measures

##### 2.1.4.1 Primary outcomes

Visual analog scale (VAS) will be accepted as the primary outcomes.

##### 2.1.4.2 Secondary outcomes

Roles and Maudsley Score (RM) will be accepted as the secondary outcomes.

### 2.2 Search methods for the identification of studies

Relevant randomized controlled trials in 6 databases (PubMed, Embase, and Cochrane Library, Chinese literature databases, Chinese Biomedical Literature Database [CBM], China National Knowledge Infrastructure [CNKI], and Wanfang Database). The RCTs of the acupotomy for calcaneodynia patients will be searched in the databases from inception to December 2017. The strategy will be created according to the Cochrane handbook guidelines. The search terms include Calcaneodynia, heel pain, talalgia, calcaneal spur, painful heel spur, plantar fasciitis, tendinitis achillea, subcalcaneal bursitis, needle knife, Small needle knife, acupotomy and randomized controlled trials. The equivalent search words will be used in the Chinese databases. The detailed strategies for searching the PubMed database will be presented in Appendix 1, and modified by using other databases.

#### 2.2.1 Searching other resources

The reference lists of studies and systematic reviews related to calcaneodynia and acupotomy will be examined for additional trials. We will also manually retrieve the relevant conference papers, and search the Clinical Trials.gov and the WHO International Clinical Trials Registry Platform (ICTRP) for new trials relevant to the topic.

### 2.3 Data collection and analysis

#### 2.3.1 Selection of studies

**Figure 1:**
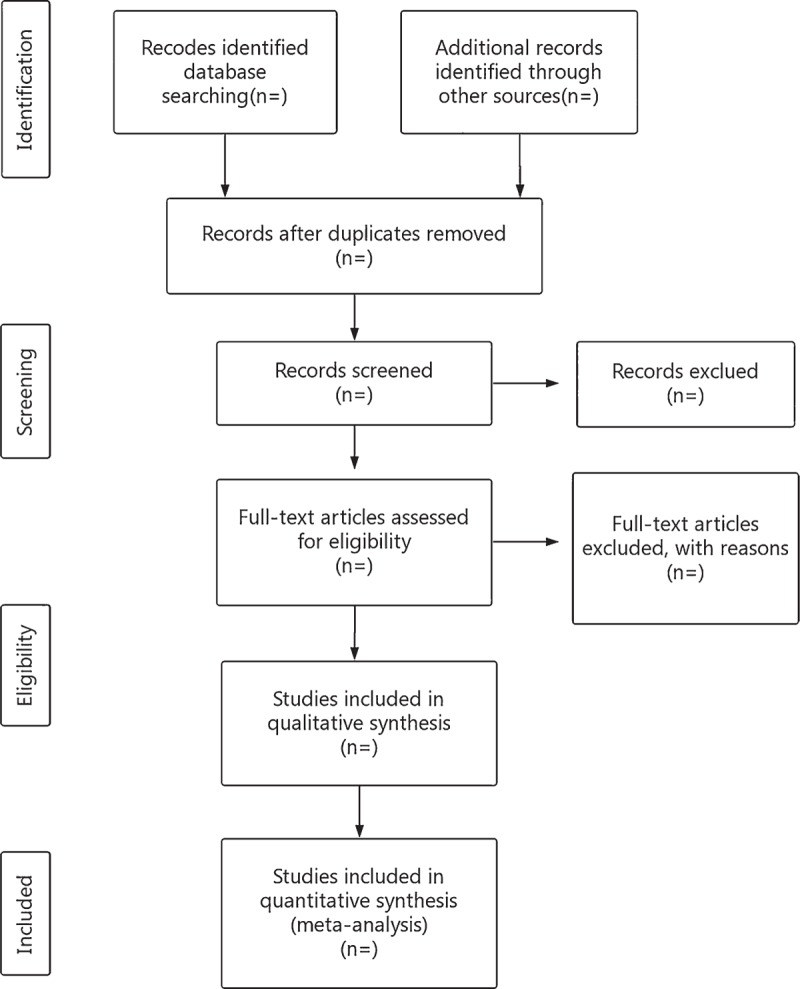
Flow diagram of study selection process.

Researchers will import the literature retrieved to the EndnoteX7 and eliminate the duplicate data. The noticeably below-standard articles will be deleted by reading the title and abstract. After that, the researchers will read the full text, discuss in the group, and contact the author for research details to determine the final inclusion of the literature (Fig. 1). The final list of articles will be converted into Microsoft Excel format. Two researchers will independently conduct the literature search and literature screening. Finally, another study member will resolve the inconsistencies and check the final literature that will be included.

#### 2.3.2 Data extraction and management

Data from the selected articles will be extracted and filled by 2 reviewers independently in the data extraction form. Any disagreement will be solved by consensus or an arbiter. We will extract information such as reference ID, author, time of publication, characteristics of participants, blinding, interventions, follow-up, outcome indicators, research results, adverse events, and other detail information. We will be in contact with the authors of trials for further information when necessary.

#### 2.3.3 Assessment of risk of bias in included studies

The risk of bias will be evaluated by 2 reviewers based on the Cochrane collaboration's tool from 7 dimensions: random sequence generation, allocation concealment, the blinding method for patients, researchers and outcomes assessors, incomplete result data, and selective reports. The risk of bias will be classified as low, unclear, and high.^[24]^

#### 2.3.4 Measures of treatment effect

The relative risk (RR) will be used to evaluate the enumeration data, the mean difference (MD) will be used to evaluate the measurement data. The effect sizes will be presented for analysis with a 95% confidence interval (95% CI).

#### 2.3.5 Dealing with missing data

We will attempt to get information by contacting the corresponding author of the referenced articles for the missing data. If the missing data cannot be obtained, we will perform our analysis based on the available data.

#### 2.3.6 Assessment of heterogeneity

The heterogeneity of the research results will be analyzed through x^2^ test (α=0.1) and determined by an I^2^ value. If I^2^≤50%, the statistic heterogeneity among trials can be negligible, and the effect size will be estimated using the fixed-effects model. If I^2^ >50%, then there is a significant heterogeneity among the trials.

#### 2.3.7 Assessment of reporting bias

When more than 10 trials are included in the study, visual asymmetry on the funnel plot will be used to determine if there is a publication bias. Quantitatively analyze with the Egger test using the software STATA 11.0 when the image is not clear.

#### 2.3.8 Data synthesis

RevMan 5.3 software will be adopted to carry out the meta-analysis. If there is no statistic heterogeneity among the results, the fixed effects model will be employed for meta-analysis. If there is a statistic heterogeneity, the source of the heterogeneity should be further analyzed. After the effect of the obvious clinical heterogeneity is excluded, the random effect model will be used for meta-analysis. If there is obvious clinical heterogeneity, the subgroup or sensitivity analysis, or only descriptive analysis can be performed.

#### 2.3.9 Subgroup analysis

If there is a significant heterogeneity in the included trials, we will conduct subgroup analysis based on the severity of calcaneodynia and types of acupotomy.

#### 2.3.10 Sensitivity analysis

When sufficient trials are available, we will perform sensitivity analysis to identify whether the conclusions are robust in the review according to the following: sample size; the effect of missing data; and methodological quality.^[25,26]^

#### 2.3.11 Grading the quality of evidence

We will evaluate the quality of evidence by the Grading of Recommendations Assessment, Development and Evaluation (GRADE) and rate it into very low, low, moderate, or high 4 levels.

## 3 Discussion

Acupotomy for calcaneodynia is a miniature surgery, with higher acceptability and less pain. It is crucial to make sure whether acupotomy is a good option for the patients, and whether it is as effective as other conservative therapies. Studies have shown that acupotomy can effectively reduce the symptoms of calcaneodynia, but its efficacy has not been evaluated scientifically and systematically. The aim of this study is to evaluate the efficacy and safety of the acupotomy treatment in patients with calcaneodynia, we hope this review will provide more evidence. There are some limitations in this review. Due to language barriers, only studies published in English and Chinese will be included. Different types of acupotomy and degree of calcaneodynia severity may run the risk of heterogeneity.^[27]^ In addition, the measurements and tools of outcomes of included studies may be different.

## Author contributions

**Data curation:** Q. Zhou, Y. Shen.

**Formal analysis:** Q. Zhou, Y. Jia, Z. Qiu.

**Investigation:** Q. Zhou, Y. Shen.

**Methodology:** Q. Zhou, Z. Qiu.

**Project administration:** S. Li.

**Software:** Y. Jia, Z. Qiu.

**Supervision:** S. Li.

**Validation:** S. Li.

**Visualization:** Y. Shen.

**Writing – original draft:** Q. Zhou, Y. Shen.

**Writing – review & editing:** S. Li.

## Supplementary Material

Supplemental Digital Content
