# Supplementary material for: Acupotomy for calcaneodynia: A systematic review protocol
Source: Medicine (Baltimore). 2018 Apr 6;97(14):e0143. doi: 10.1097/MD.0000000000010143 (PMC5902302; doi:10.1097/MD.0000000000010143)
Supplement: Supplemental Digital Content [file medi-97-e0143-s001.doc]

**Appendix 1. Search strategy used in PubMed database**

#1 ((((((((Calcaneodynia) OR heel pain) OR talalgia) OR calcaneal spur) OR sub-calcaneal fat pad lesion) OR Plantar fasciitis) OR tendinitis achillea) OR Subcalcaneal bursitis) OR painful heel spur

#2 ((acupotomy) OR small needle-knife) OR needle knife

#3 (((random[Text Word] OR randomized[Text Word]) OR control[TextWord]) OR controlment[Text Word]) OR trial[Text Word] AND "humans"[MeSH Terms]

#1AND#2AND#3
